# A Case of Lepromatous Leprosy Presenting With Multiple Morphologies in Philadelphia

**DOI:** 10.7759/cureus.76375

**Published:** 2024-12-25

**Authors:** Margaret A O'Brien, Jordan Hyde, Sylvia Hsu

**Affiliations:** 1 Dermatology, Temple University Hospital, Philadelphia, USA

**Keywords:** hansen, leprae, lepromatous leprosy, leprosy, mycobacteria, mycobacterium, mycobacterium leprae

## Abstract

Leprosy is an uncommon chronic mycobacterial infection in the United States caused by *Mycobacterium leprae*. There are two major forms of the infection, lepromatous leprosy and tuberculoid leprosy, with borderline forms of each. Leprosy is even more uncommon in the Northeastern United States and can present with various symptoms and skin findings, including erythematous or hypopigmented patches or plaques with accompanying hypoesthesia or anesthesia, anhidrosis, or alopecia. We present the case of a 29-year-old woman with a progressive rash with multiple morphologies accompanied by neuropathy and pain. Skin biopsy demonstrated acid-fast bacilli, and a diagnosis of lepromatous leprosy was confirmed. With leprosy cases on the rise, it is paramount that clinicians consider this diagnosis so that prompt treatment can be initiated. This case provides a clinical example of multiple morphologies of leprosy infection in a clinical setting where the prevalence of leprosy is very low.

## Introduction

Leprosy, or Hansen's disease, is a chronic bacterial infection caused by *Mycobacterium leprae*, a slow-growing, acid-fast bacillus with a predilection for replication in macrophages, endothelial cells, and Schwann cells. Based on the clinicopathologic findings, leprosy infection can be divided into lepromatous leprosy with a predominantly humoral response, tuberculoid leprosy with a predominantly cell-mediated response, and three borderline forms. The cardinal signs of leprosy are (1) loss of sensation in a hypopigmented or reddish plaque, (2) thickened or enlarged peripheral nerves with associated loss of sensation or muscle weakness, and (3) the presence of acid-fast bacilli in slit-skin smear. The main histopathological difference between lepromatous and tuberculoid leprosy is the presence or absence of organisms in the dermis. However, the morphology can vary, even within the same patient, as demonstrated in this case.

## Case presentation

A 29-year-old woman from Haiti presented with a diffuse rash of different morphologies of five months duration that began when she was seven months pregnant. She endorsed increasing difficulty walking, decreased sensation in her bilateral lower extremities over the previous month, subjective fevers, and a progressive intermittently painful rash of the lower extremities. She denied any other family members or close contacts with a similar rash. 

**Figure 1:**
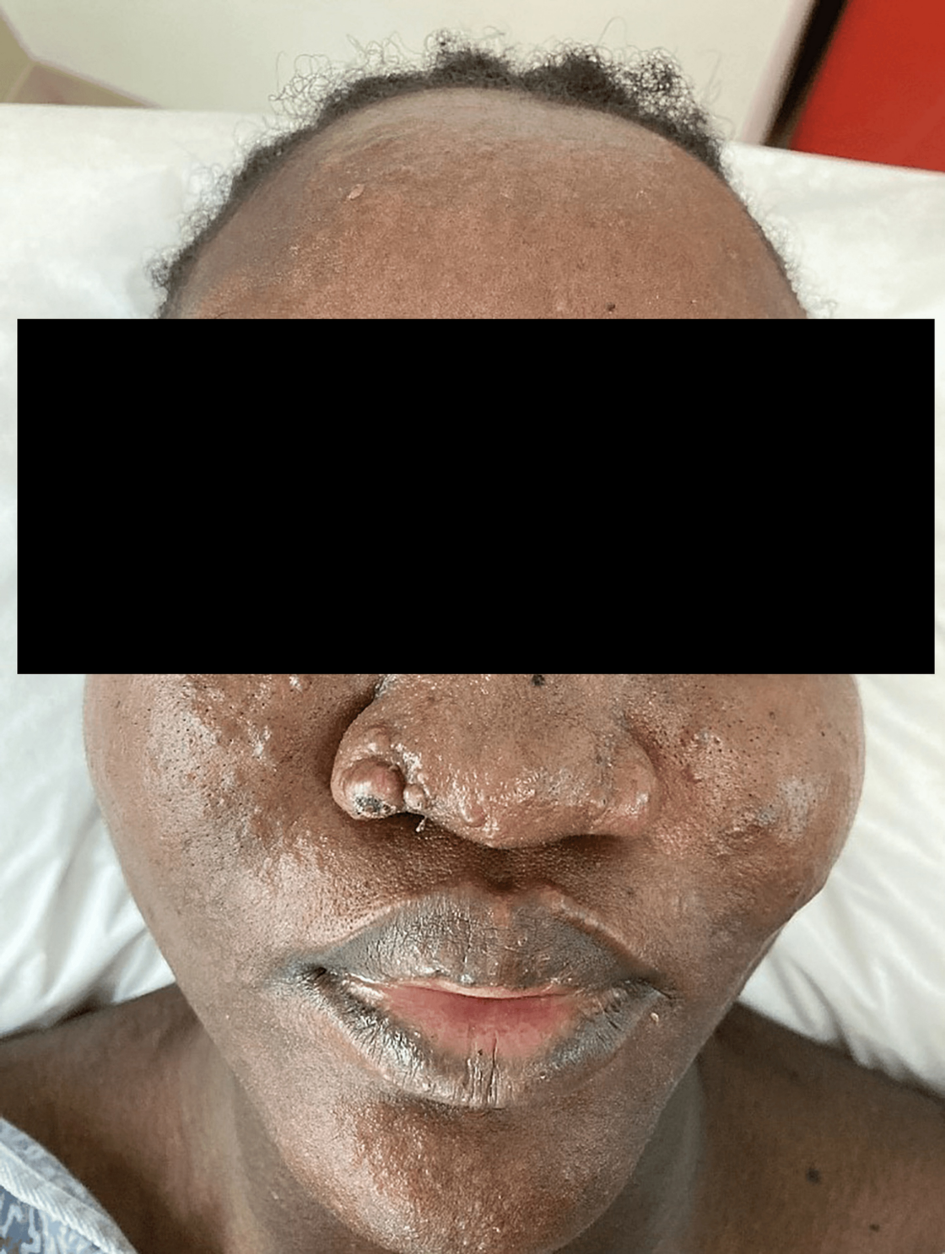
Multiple flesh-colored papules and coarse infiltration on the face over the nose, cheeks, lips, and chin.

**Figure 2:**
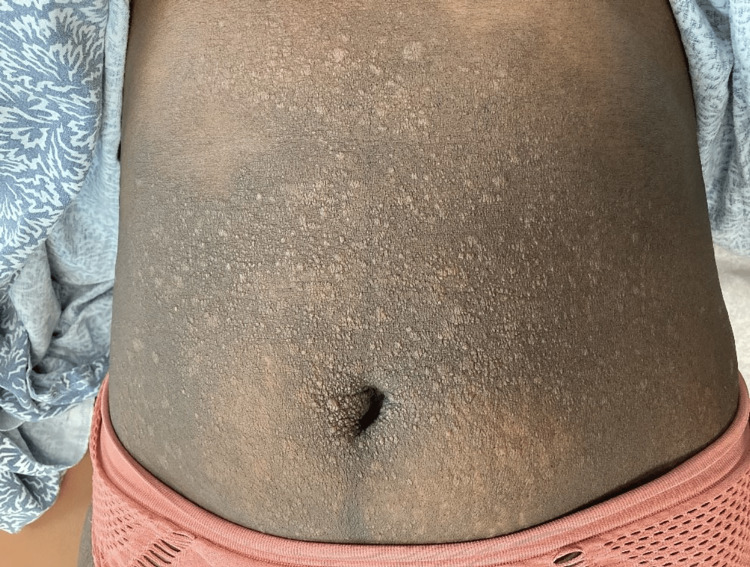
Diffuse skin-colored papules coalescing into plaques on a background of hyperpigmentation on the abdomen.

**Figure 3:**
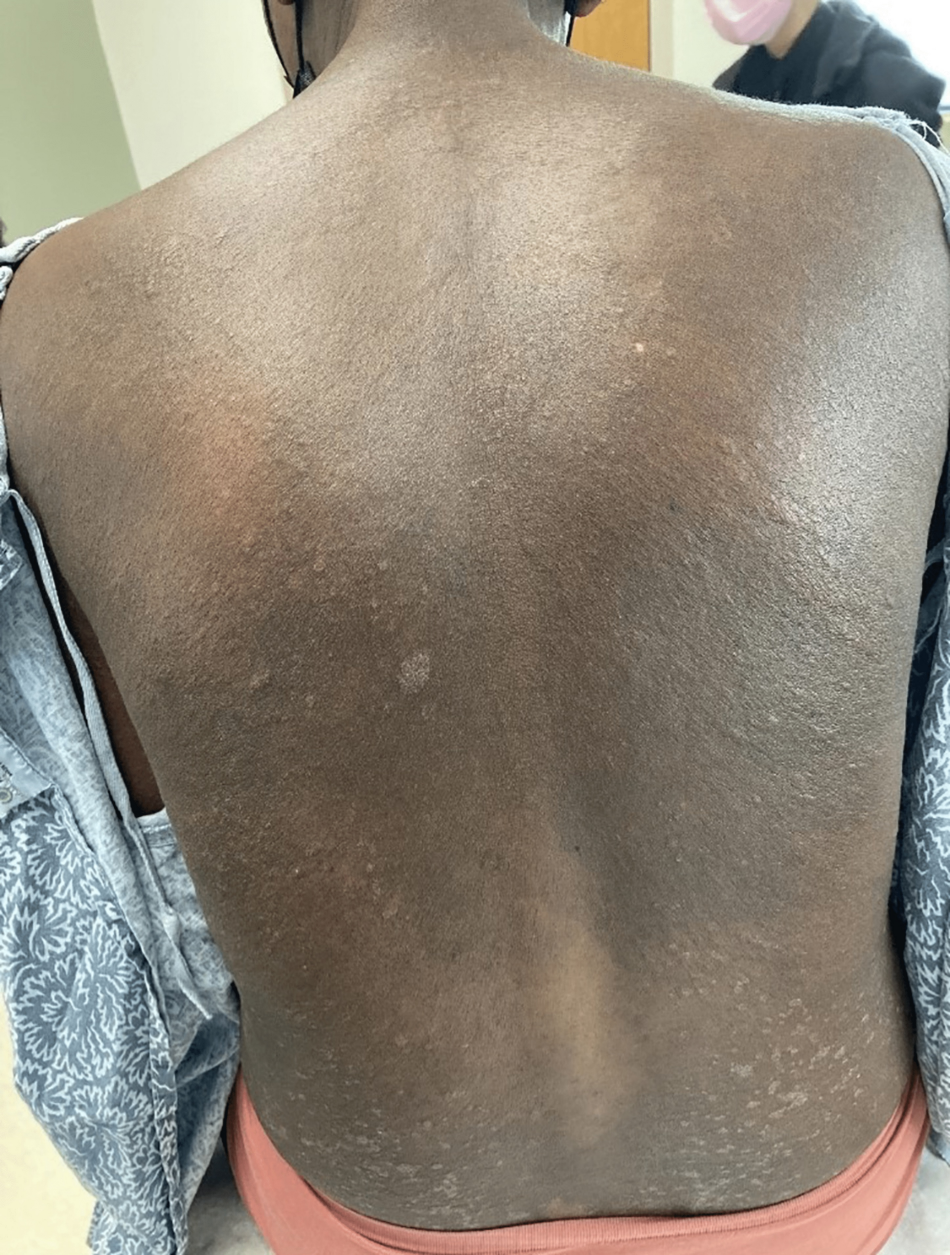
Diffuse skin-colored papules coalescing into plaques on a background of hyperpigmentation on the back.

**Figure 4:**
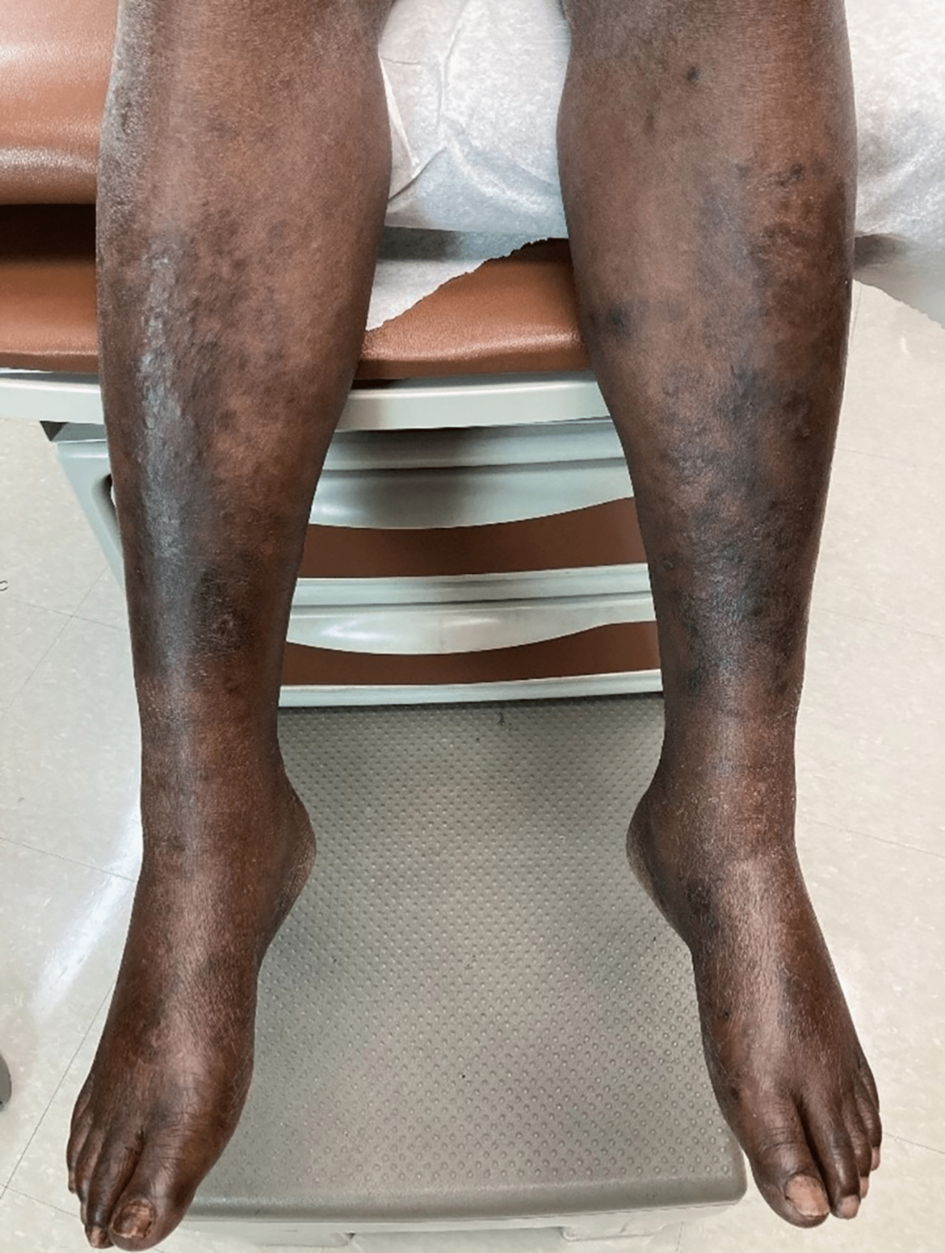
Hyperpigmented patches on the bilateral lower extremities with non-pitting edema.

She presented to our clinic with multiple skin-colored papules and nodules on her face, skin-colored papules on confluent hyperpigmented patches on the trunk, and hyperpigmented patches on the bilateral lower extremities with non-pitting edema (Figures 1-4). We performed a shave biopsy of the right nasal ala and punch biopsies of both the abdomen and the back.

**Figure 5:**
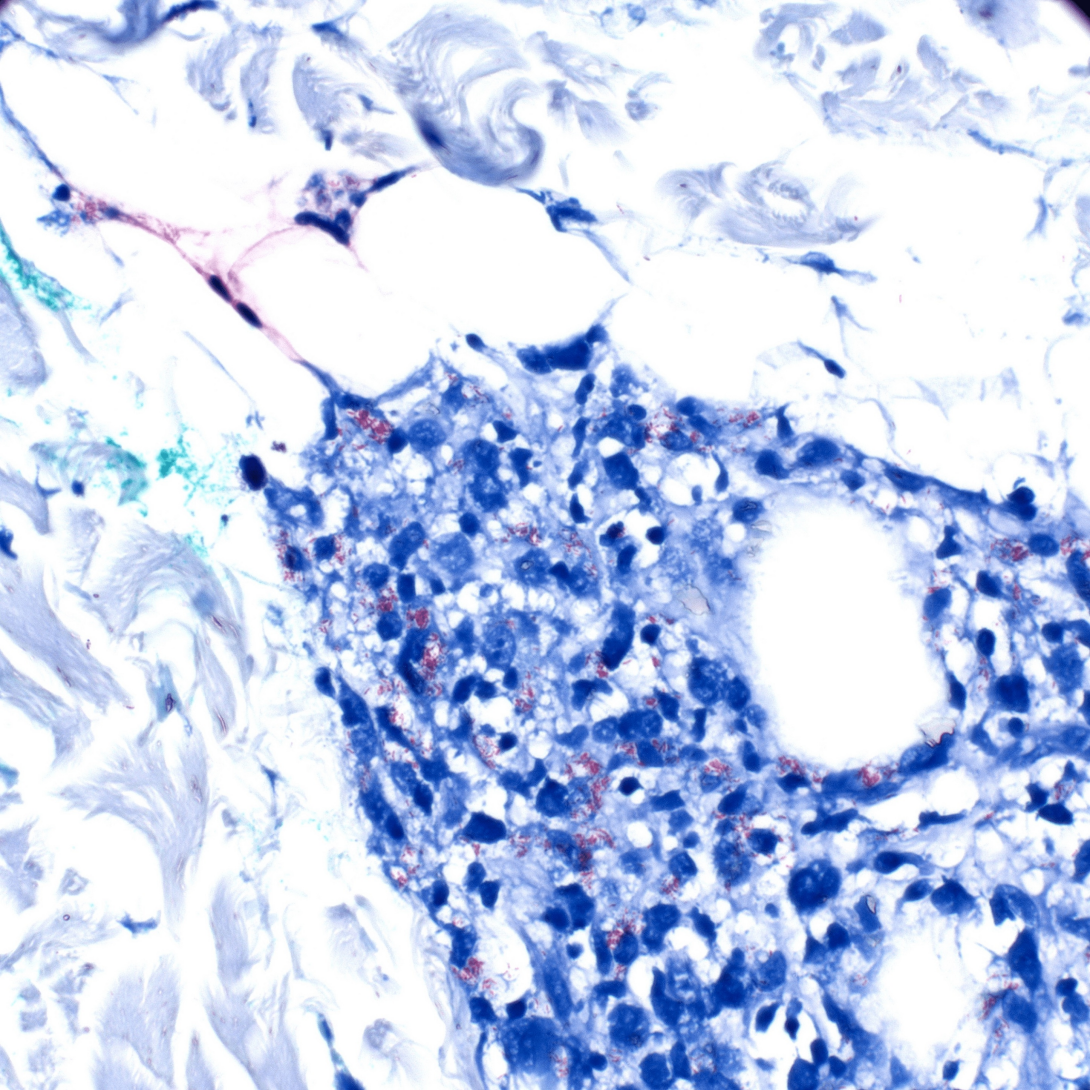
Acid-fast, rod-shaped bacilli on Fite stain (600×).

Histopathology demonstrated granulomatous inflammation and many acid-fast, rod-shaped bacilli on Fite stain, consistent with *Mycobacterium* species (Figure 5). This presentation and pathology correlate with lepromatous leprosy. The patient was then registered with the National Hansen's Disease Clinical Center, referred to infectious disease, and was started on monthly rifampin 600 mg, ofloxacin 400 mg, and minocycline 100 mg.

## Discussion

In 2000, the World Health Organization (WHO) declared leprosy "eliminated" by reaching a predefined goal of one case per 10,000 population [1]. However, the disease is far from eradicated, which is defined by a global incidence of zero. In 2023, the global prevalence of leprosy was 182,815 cases with North and South America contributing a combined 24,773 newly detected cases. The number of new cases has been steadily increasing since 2020, likely impacted by the COVID-19 pandemic [2]. In 2023, the United States reported 369 patients with leprosy, a dramatic increase from the previous year's total of 136 patients [3]. Data published by the US Department of Health and Human Services and the National Hansen's Disease Program shows that from 2004 to 2013, Pennsylvania had only 24 reported cases, representing 1.35% of total cases reported in the United States [4]. A similar patient had presented to another Philadelphia-based hospital system in 2019 [5].

Our case demonstrates the many different morphologies and symptoms that can occur with leprosy. Our patient had numerous papules on a diffuse background of hyperpigmentation on her abdomen and upper back, diffuse and symmetric hyperpigmentation of her bilateral lower extremities, and numerous skin-colored papules on her face. While hypoesthesia or anesthesia of individual lesions is a well-known finding in leprosy, it is essential to remember that lepromatous leprosy may present with relatively acute symmetric polyneuropathy in "glove and stocking" distribution [6]. The incidence of this finding in lepromatous leprosy is not known but is an important finding that can assist clinicians in making the diagnosis. It is important to recognize leprosy clinically as this is a curable disease that can progress and lead to significant morbidity without treatment.

The histology varies depending on the form of leprosy. Histologic findings in tuberculoid leprosy classically demonstrate tuberculoid granulomatous inflammation that may infiltrate nerves and have few to no organisms present. In lepromatous leprosy, histologic findings show more diffuse histiocytic infiltrate with Virchow cells (histiocytes with foamy cytoplasm) and numerous intracellular organisms that can be seen using acid-fast bacteria stains, as was the case with our patient [6].

The treatment of leprosy varies widely according to global or national protocols and therapy goals for those with smaller versus larger bacterial loads. For multibacillary leprosy, the current WHO guidelines recommend rifampicin 600 mg once a month, dapsone 100 mg daily, and clofazimine 300 mg once a month and 50 mg daily for 12 months [7]. Given the requirement for an investigational new drug (IND) application through the Food and Drug Administration (FDA) for the use of clofazimine and its risk of skin pigmentation, minocycline can be used in its place. Minocycline can also be used in place of dapsone to avoid rare but serious reactions like hemolytic anemia and agranulocytosis. Both minocycline and moxifloxacin are highly bactericidal against *Mycobacterium leprae*. The National Hansen's Disease Program recommends treatment for 24 months with dapsone 100 mg daily, clofazimine 50 mg daily, and, for those not on prednisone, 600 mg rifampin daily and, for those on prednisone, 600 mg rifampin per month [8]. Due to the long treatment duration, difficulty procuring clofazimine in the United States, and daily pill burden, once-monthly "ROM" (rifampin 600 mg, ofloxacin 400 mg, and minocycline 100 mg) therapy has become popular for borderline and lepromatous forms of leprosy. The initial studies comparing multidrug therapy (MDT) versus ROM therapy found the pulsed monthly ROM dosing to be safe and as effective as daily MDT therapy [9-11]. For these reasons, the National Hansen's Disease Program advised our patient to start this monthly ROM therapy instead of their preferred regimen.

## Conclusions

Since the prevalence of leprosy is low in the United States, especially in the Northeastern United States, it may not be included in a clinician's initial differential diagnosis. However, an astute clinician should still consider the diagnosis for a patient with an appropriate history and physical exam, even if a patient presents with various morphologies as our patient did. Furthermore, leprosy remains a public health concern as significant morbidity may occur without treatment and as cases continue to increase. As discussed, multidrug treatment regimens are variable depending on the country, availability of medications, and practicality of administration. The National Hansen's Disease Program is an excellent resource for treatment guidance within the United States. By sharing our case of lepromatous leprosy, we hope to remind clinicians about the various morphologies and presentations of leprosy. With this in mind, we can decrease the morbidity of leprosy and get closer to definitively eradicating this disease.
